# Cotton Rat (*Sigmodon hispidus*) Signaling Lymphocyte Activation Molecule (CD150) Is an Entry Receptor for Measles Virus

**DOI:** 10.1371/journal.pone.0110120

**Published:** 2014-10-08

**Authors:** Thomas Carsillo, Devra Huey, Amy Levinsky, Karola Obojes, Jürgen Schneider-Schaulies, Stefan Niewiesk

**Affiliations:** 1 Department of Veterinary Biosciences and Center for Microbial Interface Biology, The Ohio State University, Columbus, Ohio, United States of America; 2 Institute of Virology and Immunobiology, University of Würzburg, Würzburg, Germany; German Primate Center, Germany

## Abstract

Cotton rats (Sigmodon hispidus) replicate measles virus (MV) after intranasal infection in the respiratory tract and lymphoid tissue. We have cloned the cotton rat signaling lymphocytic activation molecule (CD150, SLAM) in order to investigate its role as a potential receptor for MV. Cotton rat CD150 displays 58% and 78% amino acid homology with human and mouse CD150, respectively. By staining with a newly generated cotton rat CD150 specific monoclonal antibody expression of CD150 was confirmed in cotton rat lymphoid cells and in tissues with a pattern of expression similar to mouse and humans. Previously, binding of MV hemagglutinin has been shown to be dependent on amino acids 60, 61 and 63 in the V region of CD150. The human molecule contains isoleucine, histidine and valine at these positions and binds to MV-H whereas the mouse molecule contains valine, arginine and leucine and does not function as a receptor for MV. In the cotton rat molecule, amino acids 61 and 63 are identical with the mouse molecule and amino acid 60 with the human molecule. After transfection with cotton rat CD150 HEK 293 T cells became susceptible to infection with single cycle VSV pseudotype virus expressing wild type MV glycoproteins and with a MV wildtype virus. After infection, cells expressing cotton rat CD150 replicated virus to lower levels than cells expressing the human molecule and formed smaller plaques. These data might explain why the cotton rat is a semipermissive model for measles virus infection.

## Introduction

Acute measles, a highly contagious disease, is caused by infection with measles virus (MV) and is associated with high morbidity and mortality. While effective live attenuated vaccines exist acute measles still accounts for roughly 170,000 deaths per year, particularly in children under the age of five in developing countries (WHO, 2008). It is believed that measles enters via the respiratory tract where it infects CD150+ lymphocytes, macrophages and dendritic cells before spreading to regional lymphoid tissues [1], [2], [3]. A viremia is thought to follow where virus is disseminated throughout the body via the bloodstream and infects epithelial and endothelial cells of multiple organs leading to clinical symptoms.

MV is an enveloped virus with a nonsegmented negative-sense RNA genome and belongs to the genus *Morbillivirus* in the family *Paramyxoviridae*. The virus has two glycoproteins, hemagglutinin (H) and fusion (F) protein, which mediate receptor binding and membrane fusion, respectively (reviewed in [4]). Differences in virulence between vaccine and wild type strains of MV rely, in part, on cellular entry through different cellular receptors (reviewed in [3]). Currently three cellular receptors for MV entry have been identified: membrane cofactor protein (MCP, CD46) [5], [6], signaling lymphocytic activation molecule (SLAM, CD150) [7] and nectin-4 (PVLR4) [8]; [9]. CD46, a regulator of complement activation, is expressed on all nucleated cells in humans, whereas CD150, a self-ligand co-stimulatory molecule, is expressed on thymocytes, activated lymphocytes, mature dendritic cells and macrophages ([3] and references therein), and nectin-4 is expressed on epithelial cells. It has been demonstrated that vaccine strains of MV use CD46 and CD150 for cellular entry while wild type strains use only CD150 [10], [11], [12]. The cellular distribution and expression of CD150 is consistent with the lymphotropism observed in wild type MV infections in vivo, whereas nectin-4 is used by the virus to leave an organism late in infection via the respiratory epithelium [13], [1]. A comparison of human CD150 with mouse CD150 (which does not act as a receptor for measles virus) demonstrated that the V region is crucial for binding to the MV hemagglutinin [14]. The V region of the mouse differed in amino acids at position 60, 61 and 63 from the human molecule. A mouse CD150 molecule with a “humanized” V region supported MV binding and entry like the human molecule.

The cotton rat (*Sigmodon hispidus*) has proven to be a semipermissive (titer of inoculum correlates with titer of output virus) small animal model for the study of MV pathogenesis. MV replicates in lung tissue, mediastinal lymph nodes and spleen and viral RNA can be found in peripheral blood lymphocytes [15], [16], [17], [18]. In addition, there are differences in viral spread and the immune suppressive capacity between wild type and vaccine strains. Similar to humans, wild type viruses spread to lymphoid organs and cause immune suppression [18]. This viral spread correlated with the use of human CD150 as a receptor in tissue culture. In contrast, a virus with a point mutation in the MV hemagglutinin (N481–> Y) used human CD46 and CD150 as receptor in tissue culture, and in vivo behaved like a vaccine virus and did not spread from lung tissue to lymphoid organs [18]. These data indicated that in cotton rats, as in humans, the use of CD150 by wildtype viruses is important for the pathogenesis of the disease. In this study we aimed to identify the cotton rat entry receptor for MV and subsequently tested whether the cotton rat CD150 molecule is an entry receptor for MV, analogous to human CD150.

## Materials and Methods

### Ethics statement

All animal experiments were approved by the Institutional Animal Care and Use Committee of The Ohio State University, in accordance with the Animal Welfare Act and the PHS policy.

### Animals

Inbred cotton rats and BALB/C mice were purchased from Harlan, Inc., as specific pathogen free according to the breeder's specification, and were maintained in a barrier system. Animals were kept under controlled environmental conditions of 20±2°C and a 12 hour light cycle. All animals were euthanized by CO_2_ inhalation. Female animals from 6 to 10 weeks of age were used.

### Cells and viruses

Vero-huCD150 and HEK 293T cells (human embryonic kidney) were grown in MEM with 10% FCS. BJAB cells (human B cell line, [19]) were grown in advanced RPMI 1640 supplemented with 10% FCS, 2 mmol/L glutamine, 50 IU penicillin and 50 mg streptomycin/L. Wild type MV strain Bilthoven [20], a clinical isolate derived from a MV patient, was grown on BJAB cells and titers were determined on Vero-huCD150 cells.

### Cloning of cotton rat CD150

Isolation of the cotton rat CD150 gene was performed using total RNA extracted from cotton rat splenocytes stimulated with 2.5 µg/mL Concanavalin A for 48 hours. To clone cotton rat CD150, sense 5′-A C/A CC C/T G/A TCAGCA A/G T/C C/A G/T/A C/T TC T/C C/A G/A-3′ and anti-sense 5-G/A TGG G/A G/A TTTG T/G C/T TCCTGGACAGA-3 degenerate primers based on the sequence homology of CD150 from various species (mouse, bovine, canine and human) were synthesized. Using these primers a 1023 bp amplicon was isolated using 5′ and 3′ rapid amplification of cDNA ends (RACE) PCR following the manufacturers recommendations (GeneRacer, Invitrogen). A full-length cDNA for cotton rat CD150 was PCR amplified using the sense 5′-GTGTGAATTCATGGATCCCAACAGGGCCCTTT-3′ and antisense 5′-GTGTCTCGAGTCAGCTCTCTGGTAGTGTCACA-3′ primers, containing EcoRI and XhoI restriction sites respectively (underlined), and was cloned into pcDNA4-HisMax (Invitrogen) using standard cloning techniques. The sequence of cotton rat CD150 was based on three independent clones and sequencing of cDNA mixtures. The human CD150 gene was removed from pCAG-huCD150 (kindly provided by Dr. Yanagi) and also cloned into pcDNA4-HisMax using EcoRI and XhoI restriction sites.

### Generation of L929 and HEK293T cells expressing cotton rat CD150

Stable cotton rat CD150 and human CD150 expressing cells lines were generated by transfecting L929 or HEK 293T cells with pcDNA4-CrCD150 or pcDNA4-HuCD150 using lipofectamine 2000. Cells were cultured for 48 hours and stained with a mouse antibody specific for human CD150 (clone A12, BD Biosciences) which cross-reacts with cotton rat CD150. Subsequently, cells were sorted by fluorescence activated cell sorting (i-Cyt Reflection cell sorter and analyzer) obtaining a 90% purity and resorted two weeks later to obtain a complete pure population of CD150 expressing cells. Cells were grown in selection medium containing 200 µg/mL zeocin.

### Generation of a cotton rat CD150 specific hybridoma

BALB/C mice were inoculated intraperitoneally with Concanavalin A stimulated cotton rat splenocytes and four weeks later were boosted with 2×10^6^ L929 cells expressing cotton rat CD150. Four days later mice were euthanized, spleens were removed aseptically and splenocytes were fused with 145-2C11 mouse myeloma cells. Hybridoma cells were cloned by limiting dilution three times and supernatant was tested for the presence of CD150 specific antibody by flow cytometry on splenocytes stimulated with 2.5 µg/mL Concanavalin A for 24 hours.

### Detection of cotton rat CD150 by flow cytometry

Lymphocytes from thymus, spleen, lymph nodes and Peyer’s patches were isolated by passage through a 100 micron sieve and washed three times in PBS/0.1% FCS. Lymphocytes and transfected cells were incubated with a cross-reactive antibody recognizing cotton rat MHC class I (W6/32) [21], or primary antibodies specific for cotton rat CD150 or the tag leader peptide (Asp-Leu-Tyr-Asp-Asp-Asp-Asp-Lys) (Xpress tag, Invitrogen). The secondary donkey anti-mouse polyclonal antibody labeled with FITC was pre-absorbed with cotton rat serum. Subsequently cells were analyzed by flow cytometry (Facscan, Becton Dickenson).

### Single cycle VSV pseudotyped with MV glycoproteins

**Figure 1:**
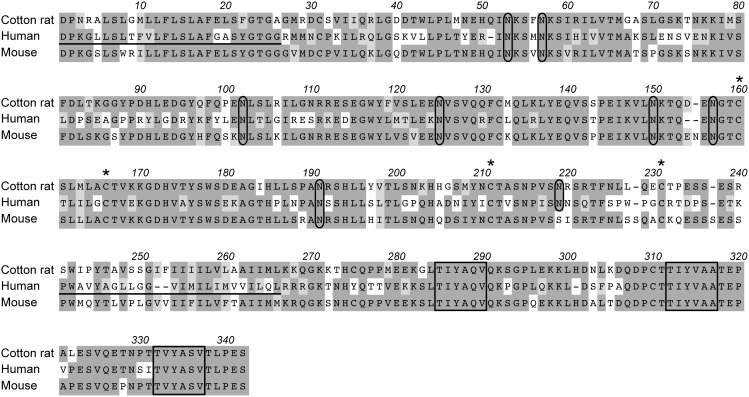
Predicted amino acid sequence alignment of cotton rat, human and mouse CD150. Spaces (indicated by dashes) denote gaps generated during alignment for optimal sequence comparison. Dark shading represents fully conserved residues, while light shading indicates conserved changes. The predicted signal peptide and transmembrane domain of human CD150 are underlined. Potential N-linked glycosylation sites are circled, four cystine residues predicted to form disulfide bonds are denoted by asterisks and three tyrosine-based switch motifs are boxed.

The use of pseudotyped VSV viruses is based on the work of Dr. Whitt ([22], see figure 1 for overview). Preparation and titration of single cycle VSV pseudotyped with MV glycoproteins were performed as described by Tatsuo et. al. with minor modifications [23]. Plasmids expressing the Edmonston vaccine strain hemaglutinin and fusion proteins, pCG-H5 and pCG-F, and the WTF wild type strain hemaglutinin and fusion proteins, pCG-WTF-H and pCG-WTF-F, were used [24]. The expression plasmid containing the cDNA for the VSV-G glycoprotein was designated pCZ-VSV-G. Briefly, 4×10^5^ HEK 293T cells were seeded in collagen coated 6-well plates (Nunc) and 12 hours later the cells were transfected with pCZ-VSV-G, pCG-H5 and pCG-F, or pCG-WTF-H and pCG-WTF-F using lipofectamine (Invitrogen). Thirty-two hours post transfection cells were infected with VSVΔG*-G at an MOI of 1 for 1 hour at 37°C. After approximately 15 to 20 hours of incubation at 37°C both the cells and tissue culture media were harvested. Following two freeze-thaw cycles, the suspension was clarified by low speed centrifugation, aliquoted and stored at –80°C. The pseudotyped viruses were denoted as VSVΔG*-G, VSV-Ed-H/F and VSV-WTF-H/F. When preparing VSV-Ed-H/F tissue culture media was supplemented with 50 µg/ml of fusion blocking peptide (Z-D-Phe-Phe-Gly) five hours post-transfection to prevent cell fusion [25]. For titration, 10-fold serial dilutions were assessed for the presence of infectious particles in a 48-well assay using HEK 293T cells for VSVΔG*-G and Vero-huCD150 for VSV-Ed-H/F and VSV-WTF-H/F. Twenty-four hours post infection cells were visualized under a fluorescent microscope and assessed for the presence or absence of GFP-expressing cells. The amount of virus in the inocula was expressed as the quantity of virus that could infect 50% of the tissue culture monolayer (TCID_50_). TCID_50_ was calculated according to the methods described by Reed and Muench [26].

### Growth curve

Growth curves were performed similar to previously described procedures [27]. 293T-CrCD150 cells and 293T-HuCD150 cells were plated in 6-well plates and infected with MV (Bilthoven strain) (MOI 0.02) and cell pellets were harvested at 0, 24, 48, and 60 hours post infection. Virus titers were quantified by TCID_50_ assay on Vero-huCD150 cells.

### Statistical analysis

The statistical analysis was performed using analysis of variance (ANOVA).

## Results

### Cloning of cotton rat CD150 cDNA

After intranasal inoculation of cotton rats (Sigmodon hispidus) with measles virus, virus can be re-isolated from lung tissue (where it infects epithelial cells and macrophages) as well as from mediastinal lymph nodes and spleen. In addition, viral RNA is detected in peripheral blood lymphocytes. The widespread infection of lymphoid cells suggested that MV uses the cotton rat homologue of human CD150 as a receptor molecule. To test this hypothesis we used rapid amplification of cDNA ends (RACE-PCR) to clone cotton rat CD150 cDNA from Concanavalin A stimulated cotton rat splenocytes using primers based on sequences from multiple species (human, mouse, bovine and canine) CD150. Cotton rat CD150 has strong homology to both human and mouse CD150 (58% and 78% homology at the amino acid level, respectively) (Figure 1). Similar to CD150 molecules from other species, the cotton rat CD150 is a type I glycoprotein with a predicted V and C2 domain. Cotton rat CD150 has eight potential N-linked glycosylation sites in common with human CD150 [28], [29], [12]. Like mouse and human CD150, cotton rat CD150 has four cystine residues predicted to form the two disulfide bonds of the C2 extracellular domain [28], [29], [12]. It also contains three immunoreceptor tyrosine-based switch motifs (ITSM), TxYxxV/I/A, in the cytoplasmic tail of the molecule which allow binding and regulation of the receptor by small SH2 adaptor proteins [12], [30]. These regions are also conserved between human, canine and bovine CD150 [12]. We also compared the amino acid residues at position 60, 61 and 63 of the V region in which the human and mouse CD150 molecule differ and which are crucial for receptor function [14]. The amino acids at position 61 (arginine) and 63 (leucine) of the cotton rat molecule are identical to the mouse molecule which is not a receptor molecule for MV. The isoleucine at amino acid position 60, however, is conserved between cotton rat and human CD150 (Figure 1). Overall, cotton rat CD150 is more similar to the mouse as compared to the human protein. However, the sequence of the measles virus binding V domain of cotton rat CD150 is more closely related to its human as compared to its mouse counterpart.

### Tissue distribution of CD150 in the cotton rat

**Figure 2:**
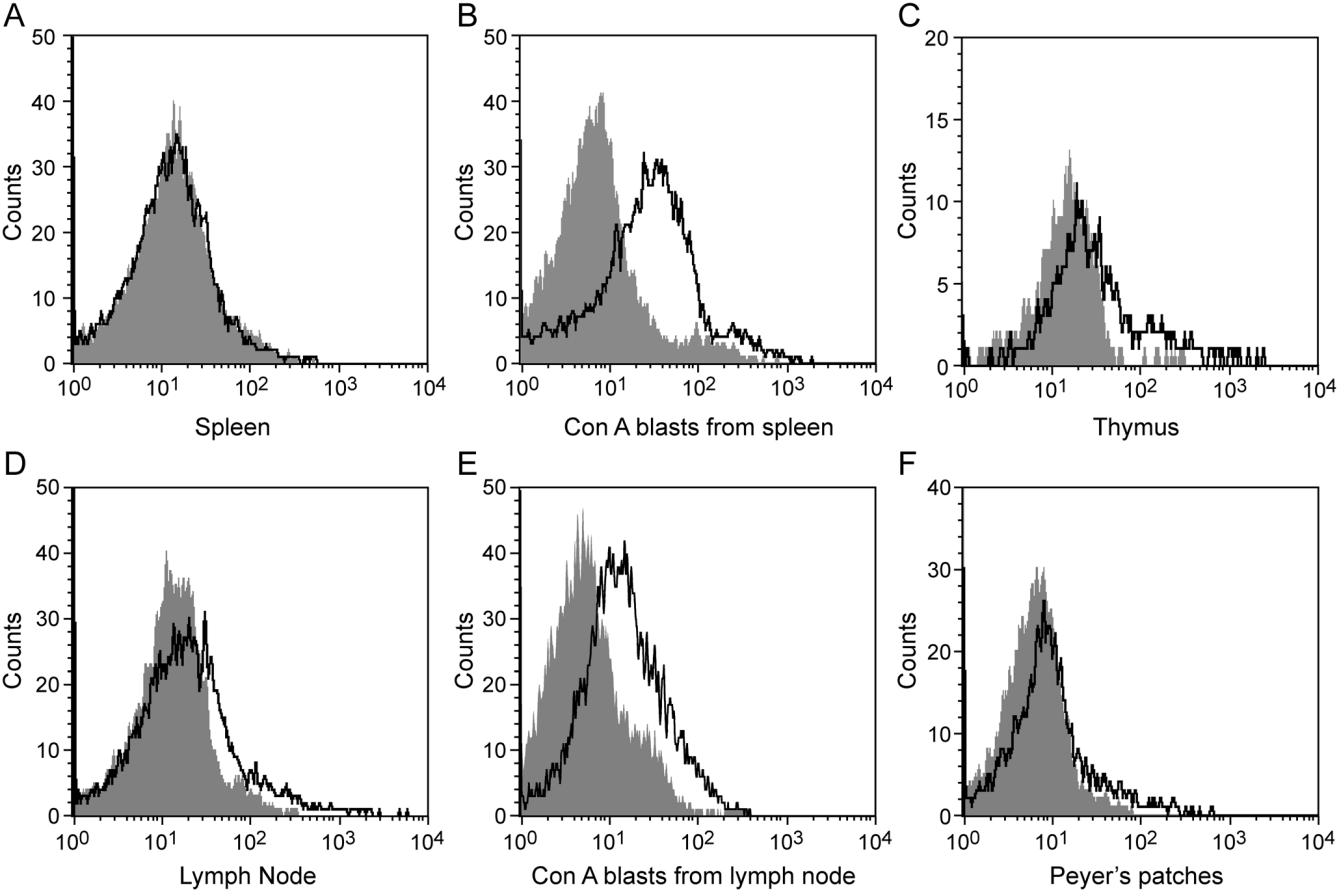
Analysis of cotton rat CD150 expression and tissue distribution. The presence of cotton rat CD150 was determined on spleen cells and lymph node cells stimulated with Concanavalin A for 24 hours (B and D), and ex vivo leukocytes from spleen (A), thymus (C), Peyer’s patches (F) and lymph node (E) (anti-CD150 black line; grey area isotype control).

In both humans and mice CD150 is expressed on a number of (activated) lymphoid cells. To determine expression of cotton rat CD150 we generated a monoclonal antibody. This antibody recognized CD150 on Concanavalin A activated spleen cells (Figure 2B) and lymph node cells (Figure 2E). CD150 was not expressed on resting ex vivo spleen cells (Figure 2A), whereas it was expressed by leukocytes from thymus (Figure 2C), lymph node (Figure 2D) and Peyer’s patches (Figure 2F). Thus the expression of CD150 correlated with the activation status of the lymphoid cells analyzed.

### Cotton rat CD150 acts as a MV entry receptor

**Figure 3:**
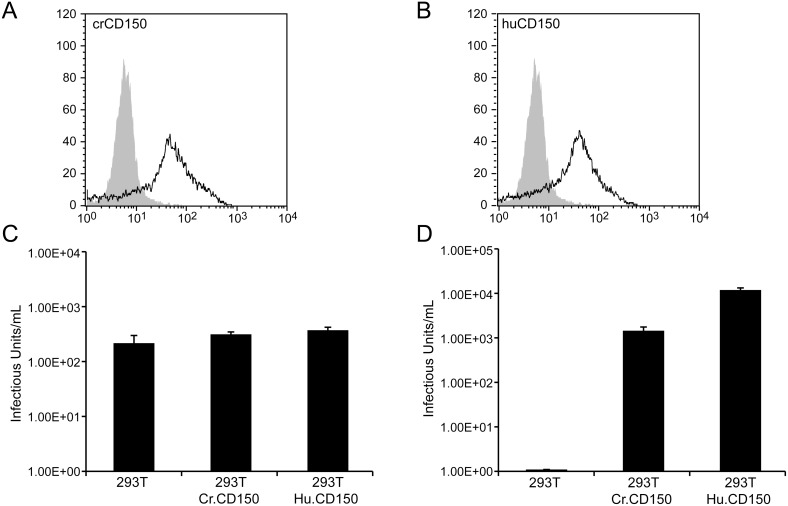
Infectivity of VSV pseudotype viruses on 293T cells expressing cotton rat and human CD150. (A) HEK 293T cells were stably transfected with plasmids expressing cotton rat (293T-CrCD150) or human CD150 (293T-HuCD150). Expression of CD150 was verified with antibodies against the cotton rat or human CD150 molecule, respectively. (B) 293T, 293T-CrCD150 and 293T-HuCD150 cells were infected with VSVΔG*-Ed-H/F. The infectivity titer was calculated by counting the number of GFP expressing cells. There was a no statistically significant difference in titer between HEK 293T, 293T-CrCD150 and 293T-HuCD150 cells. (C) 293T, 293T-CrCD150 and 293T-HuCD150 cells were infected with VSVΔG*-WTF-H/F. The infectivity titer was calculated by counting the number of GFP expressing cells. There was a significant increase in titer between HEK 293T, 293T-CrCD150 and 293T-HuCD150 cells (p<0.001, ANOVA).

To test the putative receptor function of cotton rat CD150 versus human CD150, expression plasmids were constructed containing a cDNA of the membrane-bound form of cotton rat or human CD150. The pcDNA4 based constructs contain an N-terminal epitope tag (pcDNA4-CrCD150). They were used to generate stable CrCD150- and HuCD150-transfected HEK 293 T cells, which due to the lack of human CD150 cannot be infected with wildtype MV. Expression of CD150 was confirmed by flow cytometry using the mouse monoclonal antibody specific for cotton rat or human CD150 (Figure 3a) and an antibody specific for the tag leader peptide (Asp-Leu-Tyr-Asp-Asp-Asp-Asp-Lys) (data not shown). To determine whether cotton rat CD150 acts as a virus binding and entry receptor we used a single cycle VSV pseudotype system expressing MV glycoproteins of either the vaccine strain Edmonston or the WTF wild type strain [14], [31], [7], [12]. The system relies on recombinant VSVΔG* in which the VSV G gene is replaced by a modified GFP gene and thus is not infectious unless the envelope proteins mediating receptor binding and membrane fusion are provided in *trans*. The infectivity of the virus bearing envelope proteins supplied in *trans* can be determined by counting the number of GFP expressing cells after a single cycle of viral replication. If complemented with a MV vaccine hemagglutinin and fusion protein, CD46 and CD150 positive cells can be infected; if complemented with a MV wild type hemagglutinin and fusion protein, CD150 positive cells can be infected. In both instances the presence of the fusion protein is required but does not affect the specificity of receptor binding. Pseudotyped virus bearing Edmonston H and F (VSVΔG*-Ed-H/F) grew to equivalent infectivity titers on HEK 293T cells as well as HEK 293T cells expressing cotton rat or human CD150 (Figure 3B). This was an expected result as the Edmonston H glycoprotein uses human CD46 as a receptor molecule and 293T cells express human CD46. As predicted VSVΔG*-WTF-H/F was able to infect 293T cells expressing CD150. It was taken up most efficiently by 293T-HuCD150 cells (Figure 3C). The uptake on 293T-CrCD150 cells was significantly higher as compared to control 293T cells (p<0.001), but approximately 10-fold less efficient as compared to 293T-Hu.CD150 expressing cells (p<0.001). These data demonstrate that cotton rat CD150 acts as an entry receptor for measles virus.

### HEK 293 T cells transfected with cotton rat CD150 take up MV but replicate it poorly

**Figure 4:**
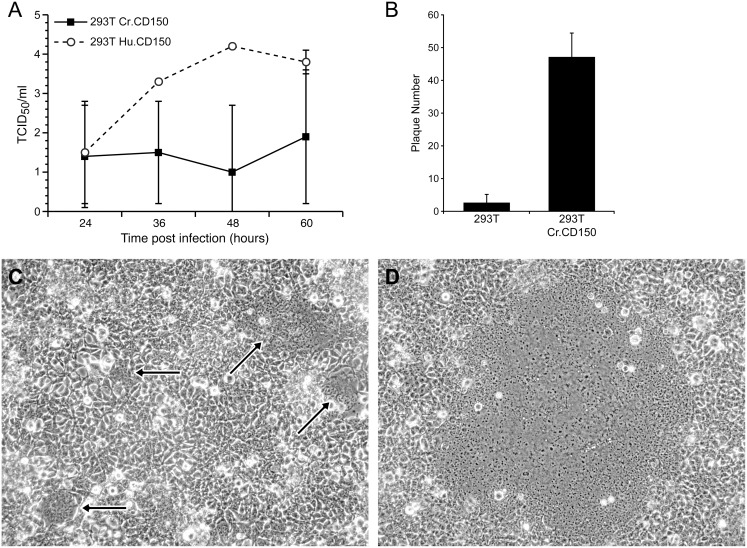
MV infection of CD150 expressing 293T cells. (A) HEK 293T and 293T-CrCD150 cells were infected with 50 pfu/well of wild type MV (Bilthoven strain) and plaques were counted 48 hours post-infection. The difference in the number of virus induced plaques was statistically significant between both cell types (p<0.001, ANOVA). (B) After infection of 293T-HuCD150 and 293T-CrCD150 with MV (Bilthoven strain) viral titers were measured at different time intervals. Infection with wild type MV (Bilthoven strain) caused the formation of numerous small viral plaques on 293T-CrCD150 cells (C) and large plaques on 293T-HuCD150 cells (D) (40x magnification).

Stably transfected 293T-CrCD150 cells were infected with 50 plaque forming units (PFU) of wild type MV (Bilthoven strain) per well of a 6-well plate. At 48 hours post infection a significant increase in viral plaque formation (47.2±7.3) was observed in transfected versus control 293T cells (2.6±2.5; p<0.001) (Figure 4A).

To compare the ability of human and cotton rat CD150 to support viral spread, we infected HEK 293 T cells stably transfected with human (293T-HuCD150) and cotton rat CD150 (293T-CrCD150). In 293T-HuCD150, viral titers peaked after 48 hours at 10^4^ pfu/ml and maintained the plateau at 60 hours, whereas in 293T-CrCD150 cells, virus was maintained only at low levels throughout the infection (Figure 4B). This difference in virus growth correlated with the production of large plaques after infection of 293T-HuCD150 cells (Figure 4D), whereas only small plaques were visible after infection of 293T-CrCD150 (Figure 4C).

## Discussion

Throughout history, morbillivirus infections have had an often devastating impact on both human and animal health. Phylogenetically, measles virus and rinderpest virus (RPV) are the two most closely related morbilliviruses. In fact, RPV is thought to be the progenitor of MV (and possibly all morbilliviruses) which was passed on to humans by domesticated cattle and subsequently evolved into MV [32]. It is also thought that RPV is the progenitor of canine distemper virus (CDV), a more distantly related morbillivirus of carnivores, and that the consumption of RPV infected prey led to the infection of carnivores and the evolution of CDV [32]. An argument supporting this evolution is the common use of CD150 of the respective species as a cellular receptor for viral entry by these three morbilliviruses [7], [12]. In addition to using the species-specific respective CD150 molecules, MV, CDV and RPV can also use their non-species counterparts as receptors, although with reduced efficiency [12], [33]. In contrast, mouse CD150 does not support MV binding and entry and this might help to explain the lack of infection by MV in the mouse [34].

The cotton rat replicates MV (both vaccine and wild type virus) in its respiratory tract and lymphoid tissue after intranasal inoculation. As in humans, viral spread is restricted for vaccine virus in contrast to wild type virus. This difference in viral spread could be reproduced using two recombinant viruses which differed in their receptor usage in tissue culture (CD46/CD150 (vaccine-like) versus CD150 (wild type-like)) [18]. These data indicated a dual receptor use (as in humans) and suggested that cotton rat CD150 might act as entry receptor. Cotton rat CD150 is a 304 amino acid long protein sharing approximately 60 and 80 percent homology to human and mouse CD150, respectively. It also shares a number of structural similarities with CD150 from other species [28], [29], [12]. The cotton rat CD150 is a type I glycoprotein with a predicted V and C2 domain and has eight potential N-linked glycosylation sites in common with human CD150, whereas the mouse molecule has seven sites [28], [29], [12]. Like mouse and human CD150, cotton rat CD150 has four cystine residues predicted to form the two disulfide bonds of the C2 domain. It also has three immunoreceptor tyrosine-based switch motifs (ITSM) in the intracellular domain of the molecule [12], [30]. In addition to its overall structure the tissue distribution of cotton rat CD150 was similar to that reported for other species, namely on activated lymphocytes and macrophages and in lymphoid tissue. Based on the sequence similarities between cotton rat, human and mouse CD150 and the tissue distribution pattern in lymphoid and non-lymphoid cells and tissue one would predict the cotton rat molecule to have the same function for the cotton rat immune system as described for the human and mouse homologs [35].

The ability of cotton rat CD150 to act as a receptor for MV was tested on transfected cell lines. Binding of MV glycoproteins and uptake was tested by infection with single cycle VSV pseudotyped with wild type glycoproteins and expressing GFP [23]. These viruses were able to infect HEK 293T cells expressing both human and cotton rat CD150, although infection of cells transfected with cotton rat CD150 was less efficient. Infection with wild type MV led to a small plaque phenotype in cells transfected with cotton rat CD150 whereas infection of cells transfected with human CD150 led to a large plaque phenotype. This may be the result of a partially reduced capacity of the CrCD150 receptor to functionally interact with MV wildtype H. This interpretation is supported by a comparative study on the human and mouse molecule [14]. Amino acids 58 to 66 within the V domain of CD150 are crucial for binding of MV-H. Within this region the mouse sequence differs in three amino acids from the human sequence at position 60, 61 and 63. Mutations within the human CD150 molecule indicate that all three positions contribute to binding of MV-H. A mouse molecule in which the valine (V) at amino acid 60 was mutated to an isoleucine (I), rendered it identical to the cotton rat molecule, is a receptor molecule with intermediate efficiency [14]. In addition, amino acids at positions 56 and 68 are different in the CD150 molecules of the three species and the amino acid leucine (L), at position 70 is the same in the human and cotton rat CD150 molecule, whereas it is a proline (P) in the mouse sequence. Since proline strongly influences the 3D structure of amino acid chains, this may also contribute to the differential receptor properties of the CD150 molecules.

Although cotton rat CD150 mediated virus entry into cells, it did not efficiently support virus replication (in contrast to human CD150). This was not only seen in HEK 293T cells but also in Vero cells transfected with either the human or cotton rat CD150. In Vero cells, cotton rat CD150 also led to a number of small plaques but not to widespread virus replication (data not shown). It has been observed previously that transfection of human CD150 into mouse or rat cells does lead to virus uptake but no virus replication. However, this phenomenon has been hypothesized to be due to lack of intracellular factors required to support virus replication. In contrast to these findings, both HEK293T and Vero cells support virus replication of the vaccine virus and a receptor molecule for the wildtype strain theoretically should mediate entry and not influence replication of the virus. As shown in Figure 3B, cotton rat CD150 supports virus-cell fusion approximately 10-fold less well than human CD150. This effect in addition to a similar reduction of cotton rat CD150 in mediating cell-to-cell spread of the virus may account for the reduced virus titter and plaque size as observed in Figure 4B and 4C. It is also possible that species specific differences make the interaction of a cotton rat molecule with primate cells (Vero) less effective; for instance, the cytoplasmic tail of cotton rat CD150 might interact less efficiently with the primate signal transduction system. Alternately, cotton rat CD150 might be less effective in supporting measles virus replication and this phenomenon might explain why the cotton rat is a semipermissive model for measles virus.
